# Genomic Characteristics and Pan-Genome Analysis of *Rhodococcus equi*

**DOI:** 10.3389/fcimb.2022.807610

**Published:** 2022-02-16

**Authors:** Yang Song, Xinmin Xu, Zhenzhou Huang, Yue Xiao, Keyi Yu, Mengnan Jiang, Shangqi Yin, Mei Zheng, Huan Meng, Ying Han, Yajie Wang, Duochun Wang, Qiang Wei

**Affiliations:** ^1^ National Pathogen Resource Center, Chinese Center for Disease Control and Prevention (China CDC), Beijing, China; ^2^ Department of Clinical Laboratory, Beijing Ditan Hospital, Capital Medical University, Beijing, China; ^3^ Center for human Pathogenic Culture Collection, National Institute for Communicable Disease Control and Prevention, China CDC, Beijing, China

**Keywords:** genomic characteristics, plasmid-mediated pathogenicity, rifamycin resistance, *Rhodococcus equi*, pan-genome

## Abstract

*Rhodococcus equi* is a zoonotic pathogen that can cause fatal disease in patients who are immunocompromised. At present, the epidemiology and pathogenic mechanisms of *R. equi* infection are not clear. This study characterized the genomes of 53 *R. equi* strains from different sources. Pan-genome analysis showed that all *R. equi* strains contained 11481 pan genes, including 3690 core genes and 602 ~ 1079 accessory genes. Functional annotation of pan genome focused on the genes related to basic lifestyle, such as the storage and expression of metabolic and genetic information. Phylogenetic analysis based on pan-genome showed that the *R. equi* strains were clustered into six clades, which was not directly related to the isolation location and host source. Also, a total of 84 virulence genes were predicted in 53 *R. equi* strains. These virulence factors can be divided into 20 categories related to substance metabolism, secreted protein and immune escape. Meanwhile, six antibiotic resistance genes (*RbpA, tetA (33), erm (46), sul1, qacEdelta 1* and *aadA9*) were detected, and all strains carried *RbpA* related to rifamycin resistance. In addition, 28 plasmids were found in the 53 *R. equi* strains, belonging to Type-A (n = 14), Type-B (n = 8) and Type-N (n = 6), respectively. The genetic structures of the same type of plasmid were highly similar. In conclusion, *R. equi* strains show different genomic characteristics, virulence-related genes, potential drug resistance and virulence plasmid structures, which may be conducive to the evolution of its pathogenesis.

## Introduction

The genus *Rhodococcus*, a group of facultative aerobic Gram-positive bacteria, belongs to nocardiaceae, Corynebacterium suborder, and is widely distributed in rocks, groundwater, soil, marine sediments, insects, animals and plants (Gao and Gupta, 2012; Vazquez-Boland et al., 2020). Among the 57 species in the genus *Rhodococcus*, at least six species (*R. equi*, *R. erythropolis*, *R. ruber*, *R. gordoniae R. fascians* and *R.defluvii*) have been proved to be related to animal and plant pathogenicity (Sangal et al., 2015; Anastasi et al., 2016; Canetti et al., 2019; Ying et al., 2019). Among these six species, *R. equi* is the main pathogen of human infection. Also, as the only opportunistic pathogen in the genus *Rhodococcus*, *R. equi* has been widely concerned by medical community (Takai et al., 2020).

The main hosts of *R. equi* are foals and humans with immunodeficiency, especially HIV-infected patients (Lin et al., 2019). In addition, pigs, cattle, sheep, cats, dogs are also occasionally infected (Bryan et al., 2017; Stranahan et al., 2018). It was reported that in humans, *R. equi* infection usually occurs in lungs, causing pneumonia and suppurative lesions. Common symptoms include fever, cough and chest pain. In addition to pulmonary infection, *R. equi* can also invade other tissues and organs, such as bronchus, kidney and brain, resulting in bronchitis, meningitis, bacteremia and other diseases. In AIDS patients, in spite of rapid diagnosis and proper treatment *R. equi* infection can lead to serious and even life-threatening diseases (Albert et al., 2021). The mortality of AIDS patients with combined *R. equi* infection was as high as 20%~58% (Ahumada et al., 2020). With the extensive application of antibiotics, reports of multidrug-resistance in *R. equi* are increasing, including resistance to trimethoprim, sulfamethoxazole, ampicillin sulbactam, clindamycin, erythromycin and rifampicin (Petry et al., 2020; Alvarez-Narvaez et al., 2021a; Huber et al., 2021).

The pathogenicity of *R. equi* depends on a variety of virulence factors, including capsular polysaccharide, lipid composition, extracellular enzyme and virulence plasmid (von Bargen and Haas, 2009; Da et al., 2020; Salazar-Rodriguez et al., 2021). An 80 ~ 90 kb virulence plasmid with host specificity is regarded as an important virulence factor of *R. equi*, and the virulence-related protein (VAP) encoded by the plasmid is regarded as the key to the survival and proliferation of *R. equi* in macrophages (Letek et al., 2008). So far, three types of plasmids have been identified: type-A, B and N, carrying specific VAPs highly related to different humans and animal hosts (MacArthur et al., 2017; Vázquez Boland and Meijer, 2019). In humans, most *R. equi* isolates carry type-B plasmids, while type-A and type-N plasmids are relatively few (Valero-Rello et al., 2015; Suzuki et al., 2021). In previous studies, strains carrying type-A plasmids were regarded as high toxic, strains carrying type-B plasmids as medium toxic, type-A and type-B plasmids negative strains as non-toxic (Jain et al., 2003). However, the three types of strains can be isolated from clinical patients and have no correlation with plasmid toxicity, suggesting that *R. equi* may infect humans through other virulence mechanisms.

In the past, it was considered that *R. equi* was a rare opportunistic pathogen of zoonosis. *R. equi* infection mostly occurred in individuals with impaired immunity, such as patients with organ transplantation, malignant tumors and human immunodeficiency virus infection. In recent years, cases of *R. equi* infection in AIDS patients have rapidly increased. Meanwhile, the prevalence of multi-drug resistance of *R. equi* posed new challenges for clinical and scientific research. With the development of sequencing technology, the reduction of sequencing cost and the increase of genomic data in public databases, it is possible to study *R. equi* by comparative genomics. In this study, the genomes of four *R. equi*　strains isolated from HIV patients were sequenced. Combined with the whole-genome sequences of 49 *R. equi*　strains from public database, we performed pan genome analysis to explore the genomic characteristics, virulence factors and antibiotic resistance of *R. equi*. Our results provide a theoretical basis for the study of the pathogenesis and drug resistance mechanism of *R. equi*.

## Materials And Methods

### Strain Information

Four strains (CHPC 1.8375, CHPC 1.8383, CHPC 1.8384 and CHPC 1.8376) were isolated from HIV patients (males, aged 24-45) hospitalization in Beijing Ditan Hospital Capital Medical University. Among them, CHPC 1.8375, CHPC 1.8383 and CHPC 1.8384 were isolated from alveolar lavage fluid, CHPC 1.8376 was isolated from bone marrow. The four strains were identified as *R. equi* by 16S rRNA gene analysis. these strains were cultured in BHI medium at 37°C, and stored in Center for Humans Pathogenic Culture Collection, Chinese CDC. These four clinical *R. equi* strains, together with 49 *R. equi* strains and 33 strains of other *Rhodococcus* spp. from GenBank (genomes with less than 200 contigs were selected) were included in this study. Detailed information of these strains was listed in **Tables S1** and **S2**.

### Genome Sequencing

The genomes of the four clinical *R. equi* isolates were extracted by Wizard Genomic DNA Extraction Kit (Madison, WI, Promega, USA) following the manufacturer’s instructions. The HiSeq sequencer (Illumina HiSeq2000, San Diego, CA, USA) was used to perform 250-bp paired-end whole-genome sequencing with 150×coverage. FastQC (http://www.bioinformatics.babraham.ac.uk/projects/fastqc/) was used to evaluate the quality of the reads (Low-quality reads were discarded if the quality scores of ≥ 3 consecutive bases were ≤ Q30). Readfq (version 10) to filter the original data to obtain effective data (clean data). SOAP *de novo* (Version 2.04) was used to assemble the clean data of each strain and finally integrate it with CISA (Contig Integrator for Sequence Assembly) (Lin and Liao, 2013; Honaas et al., 2016).

### Gene Variation Detection

SAMtools was used to detect the SNPs, small fragment insertion and deletion (InDel) with a length of less than 50 bp of the 4 clinical strains, and the variation of SNP/InDel in the functional region of the genome was analyzed. Burrows-Wheeler-Alignment (BWA) was used to compare the reads to the reference sequence 103S, and SAMtools was used to interpret the comparison results and count the coverage of reads to the reference sequence 103S (Li and Durbin et al., 2010). Circos was used to visualize the coverage of reads and the distribution of SNP and InDel (Krzywinski et al., 2009).

### Average Nucleotide Identity (ANI) Analysis

The evolutionary distance of 37 *Rhodococcus* spp. strains was evaluated at the genomic level by ANI analysis. These strains included 4 newly sequenced clinical *R. equi* isolates and 33 reference *Rhodococcus* strains from Genebank. ANI values were calculated by JSpecies software. Strains with ANI value > 95% were considered as the same species (Lee et al., 2016).

### Pan-Genome Analysis

The genomes of 53 *R. equi* strains were collected together as a local database. Roary and BPGA software were used for pan-genome analysis to obtain the pan-genome information of *R. equi* (Page et al., 2015; Chaudhari et al., 2016). The pan-genome sequences were then compared with the COG database by NCBI blast + software to obtain COG annotation information (cutoff value of ≥ 90% sequence identity and ≥ 60% length coverage).

### Phylogenetic Analysis

Considering that there may be differences in pathogenic genes between clinical and environmental strains of *R. equi*, after the extraction of pan-genome sequence of *R. equi*, phylogenetic trees were constructed with the pan-genome dataset based on Bayesian Inference. The CIPRES Science Gateway V 3.3 was used to perform MrBayes analysis, MrBayes software (version 3.2.2) was used to construct a phylogenetic evolutionary tree and setting GTR + invgamma, 107 generations, sampling every 1000 generations with a burnin fraction of 0.25 (Altekar et al., 2004; Miller et al., 2015).

### Virulence-Related Genes Analysis

The Virulence Factor Database (VFDB) was used to predict the virulence-related genes of *R. equi*. An automatic analysis pipeline was provided by VFanalyzer (Liu et al., 2019).

### Antimicrobial Resistance Genes Analysis and *In Vitro* Susceptibility Testing

The CARD (Comprehensive Antibiotic Research Database) was used to predict the potential antibiotic resistance genes of *R. equi* (Jia et al., 2017). The parameters of BLAST+ were an E-value of 1e-5, a percentage of sequence identity ≥ 80% and a percentage of length coverage ≥ 80%. Detailed information of the antimicrobial resistance genes was listed in **Table S3**. *In vitro* susceptibility testing was determined by micro broth minimum inhibitory concentration (MIC) method, the MIC values were obtained by analysis, and the results of sensitivity, moderate sensitivity, and drug resistance were obtained according to the corresponding standards of the American Committee for Clinical Laboratory Standardization (CLSI) (Clinical and Laboratory Standards Institute, 2014), *E. coli* ATCC 25922^T^ was used as a quality control.

### Plasmid Related Gene Analysis

Gathering the plasmid genes of 20 *R. equi* strains as a local database, the plasmid genes were annotated by Prokka (version 1.12) and standards-compliant *.gff output files were produced for each sample. The pan-genome of the plasmid sequences was obtained by Roary. PhyML (version 3.1) was used to construct a phylogenetic tree by maximum likelihood method. ACT (Artemis Comparison Tool) was used for gene collinearity analysis (Abbott et al., 2005).

## Results

### General Features of *R. equi* Genomes

**Figure 1:**
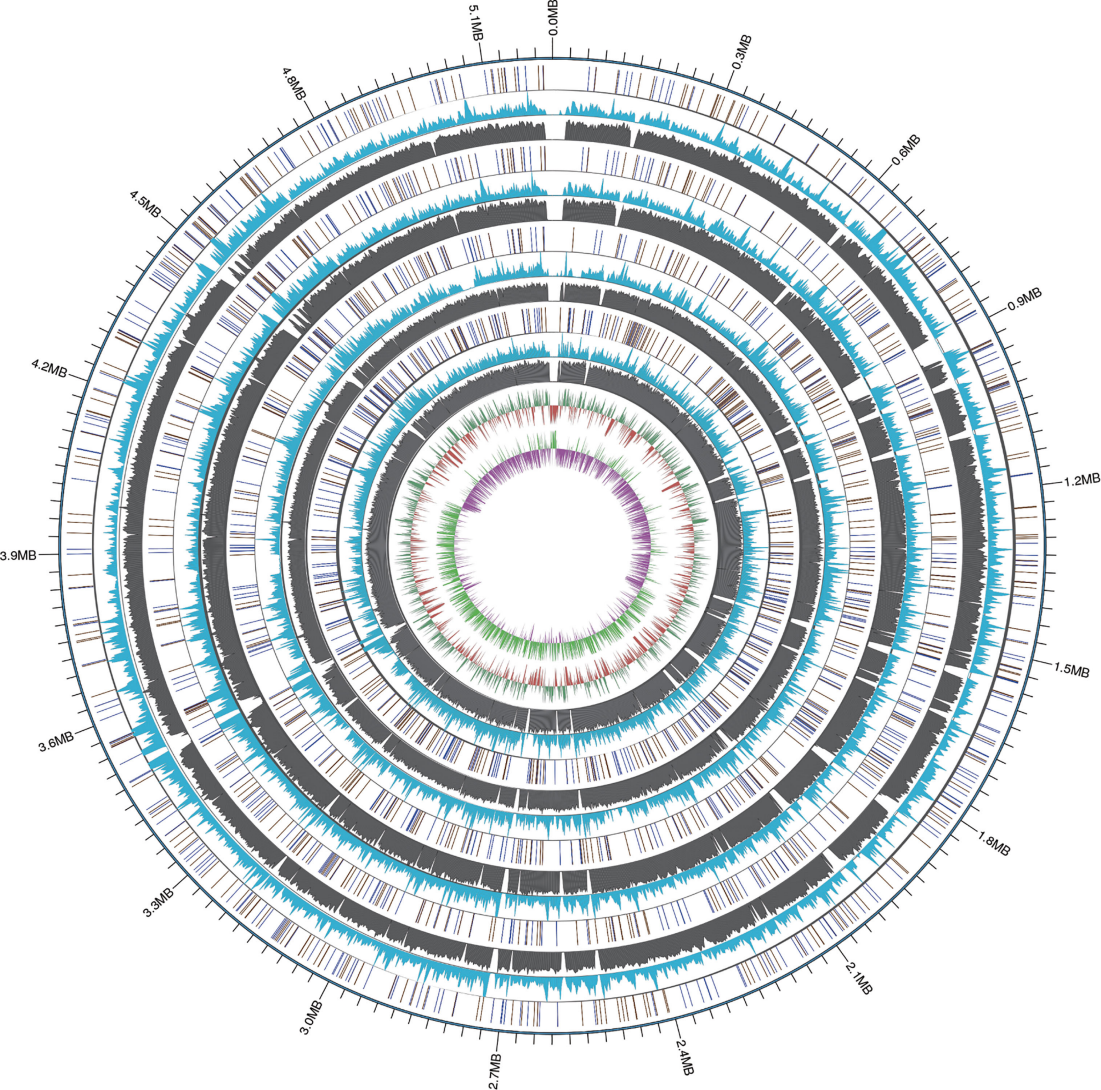
The WGMP (Whole Genome Mutation Profile) of 4 clinical isolates in this study. The outermost circle of the picture was the position of gene correspond to the reference genome (NCBI accession number: GCA_016025875.1). From the outside to the inside, InDel distribution, SNP number distribution, reads coverage depth, GC content of the reference sequence genome and GC skew value distribution of the 4 clinical isolates were shown. In the InDel distribution, the color of blue indicated deletion, yellow indicated insertion.

The genome size of 53 *R. equi* strains was 5.24 ± 0.14Mb, and the average GC content was 68.72% (**Table S1**). Among the 4 clinical isolates of *R. equi* genome newly sequenced in this study, the genome size was between 5.08 and 5.26 Mb, and the content of GC ranged from 68.66% to 68.78%. The CDS of the genome of the 4 clinical *R. equi* isolates was between 4710 and 4937, while the rRNA was between 3 and 8 and the tRNA ranged from 60 to 66. All strains contained only one tmRNA. Also, the 4 clinical *R. equi* strains newly sequenced in this study carried type-B plasmids. The single nucleotide polymorphism (SNP) of the 4 strains was distributed between 34592 and 37870, and the conversion transversion rate was 1.47% ~ 1.48%, the SNP were similar among sequencing strains (**Figure 1**).

### Average Nucleotide Identity (ANI) Analysis

**Figure 2:**
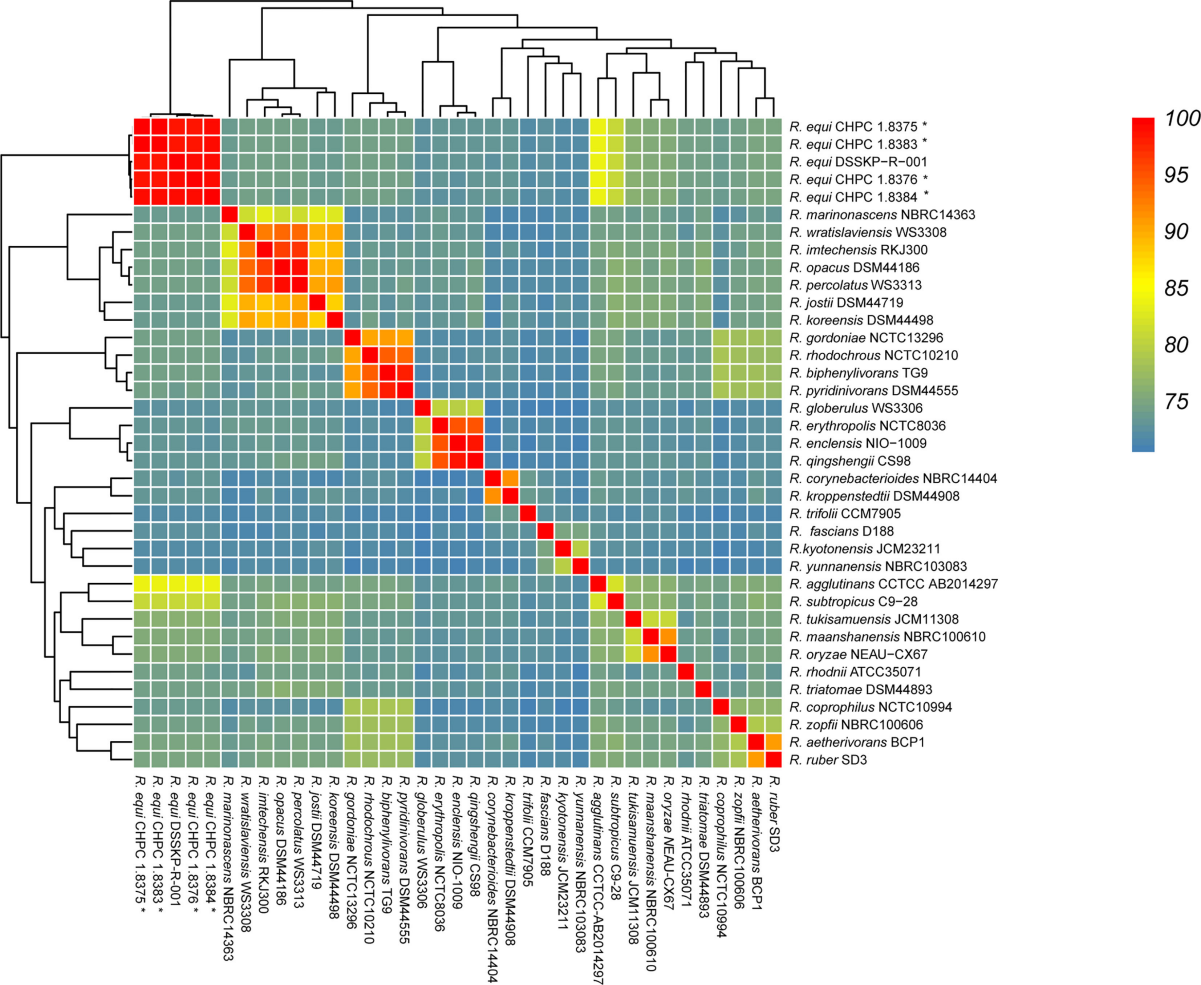
Average Nucleotide Identity (ANI) analysis of the genomes of 37 *Rhodococcus* strains, the strains sequenced in this study were marked with *.

The average nucleotide identity (ANI) analysis based on the whole genome confirmed that the four clinical isolates newly sequenced in this study belonged to *R. equi* (CHPC 1.8375 ANI value 98.71%, CHPC 1.8376 ANI value 99.12%, CHPC 1.8383 ANI value 98.75%, CHPC 1.8384 ANI value 99.02%, DSSKP-R-001 ANI value 100%, higher than the cut off value of ANI species level, 95%). The results of ANI analysis showed that in the strains of *Rhodococcus* spp., *R. agglutinans* (ANI 83.6% ~ 83.8%) and *R. subtropicus* (ANI 81.0%) have the highest nucleotide consistency with *R. equi*. The ANI values of other *Rhodococcus* spp. strains were less than 80%, indicating that they have great differences from *R. equi*. The ANI analysis also calculated the similarity of each pair of genomes of different *Rhodococcus* spp., and the ANI value ranged from 70.51% to 100%, the ANI values of intraspecific was higher than interspecific (**Figure 2**).

### Pan-Genome Analysis

**Figure 3:**
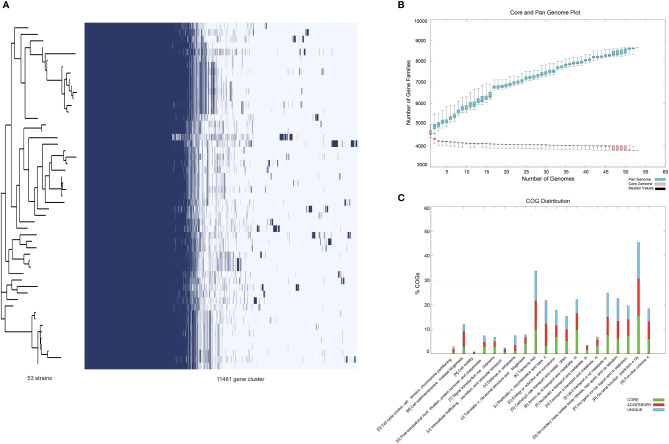
Pan-genomic analysis of 53 *R. equi* strains. **(A)** Pan-genomic distribution. The tree on the left represented the evolutionary relationship based on Pan-genome, while the figure on the lift represented the presence and absence of core and accessory genes. **(B)** Number of gene family. **(C)** The COG distribution. Green color represented core gene, red and blue represented accessory gene and unique gene, respectively.

The pan-genome of the 53 *R. equi* strains contained 11481 pan genes, including 3690 core genes and 602 ~ 1079 accessory genes (**Figure 3A**). When the number of the genomes of *R. equi* strains increased, the total number of gene families increased and the number of core genes tended to be consistent, indicating that *R. equi* has an open pan-genome (**Figure 3B**). The COG enrichment analysis of the pan-genome showed that the 11481 pan genes were classified into 20 functional categories; the predominant category was the cluster of metabolism, followed by information storage and processing, cell biological processes and signal pathways. A number of cores were assigned to cluster of general function prediction only, transcription, amino acid transport and metabolism. Whereas, a large number of accessory genes were assigned to general function prediction only, transcription and replication, recombination and repair. Unique genes were assigned to general function prediction only, transcription, lipid transport and metabolism (**Figure 3C**).

### Phylogenetic Analysis

**Figure 4:**
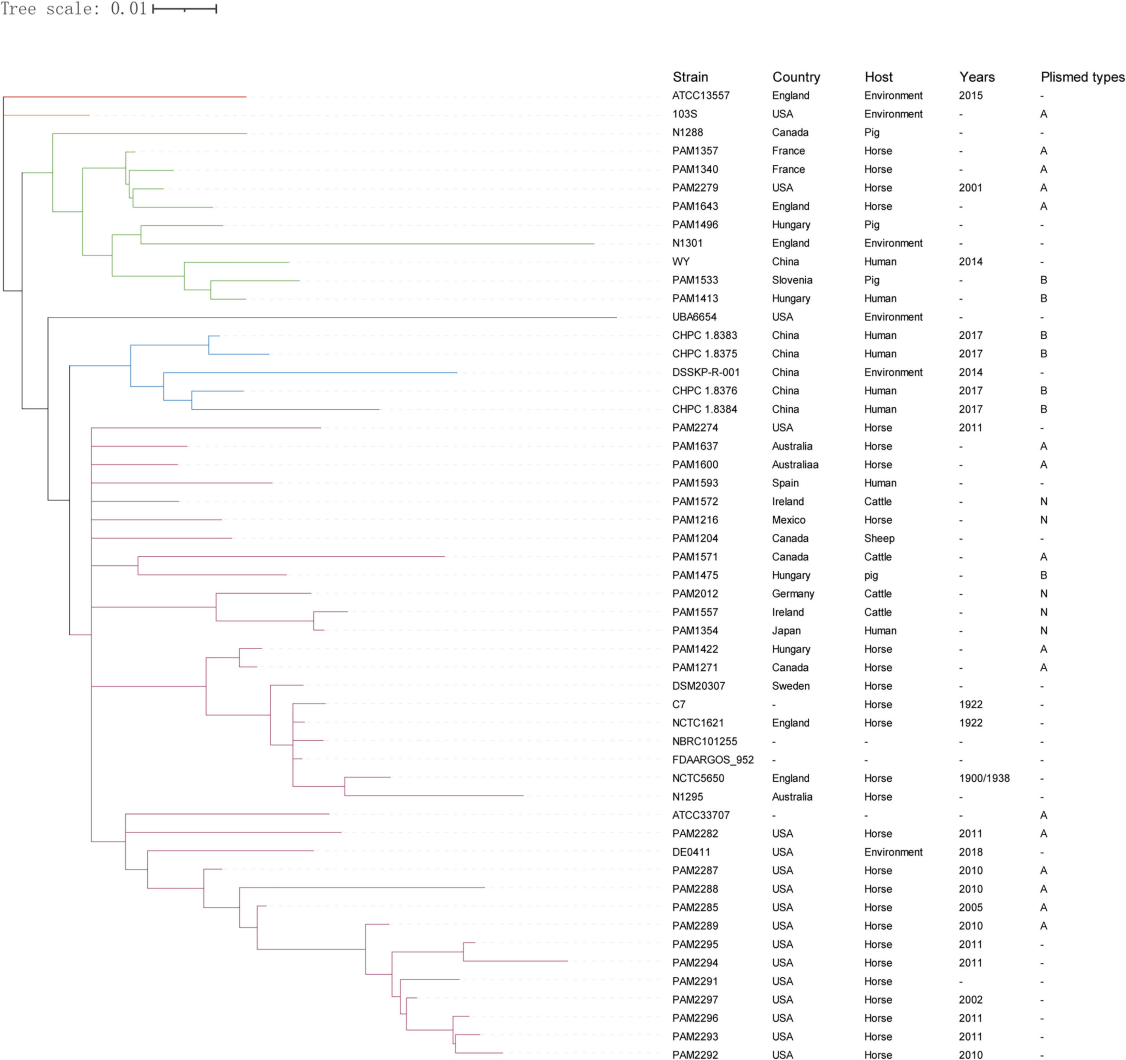
Phylogenetic analysis of the pan- genome sequences of 53 *R. equi* strains. The 53 strains were divided into six clades and different clades were labeled in different colors. The isolated country, host source, isolated year and plasmid types were shown on the right. “-” means not collected.

Phylogenetic analysis based on the pan-genome of the 53 *R. equi* strains showed that these strains were clustered into six clades, which was not directly related to the isolation location and host source. The 4 clinical *R. equi* isolates newly sequenced in this study did not show any obvious genetic evolution relationships with other *R. equi* strains isolated from humans, even the clinical strain WY from China in 2014. However, the 4 clinical *R. equi* isolates newly sequenced in this study were in the same cluster with the environmental strains DSSKP-R-001 isolated from China in 2014, which suggests that the 4 clinical *R. equi* isolates may from patients infected by *R. equi* from the environment (**Figure 4**).

### Virulence-Related Genes Analysis

**Figure 5:**
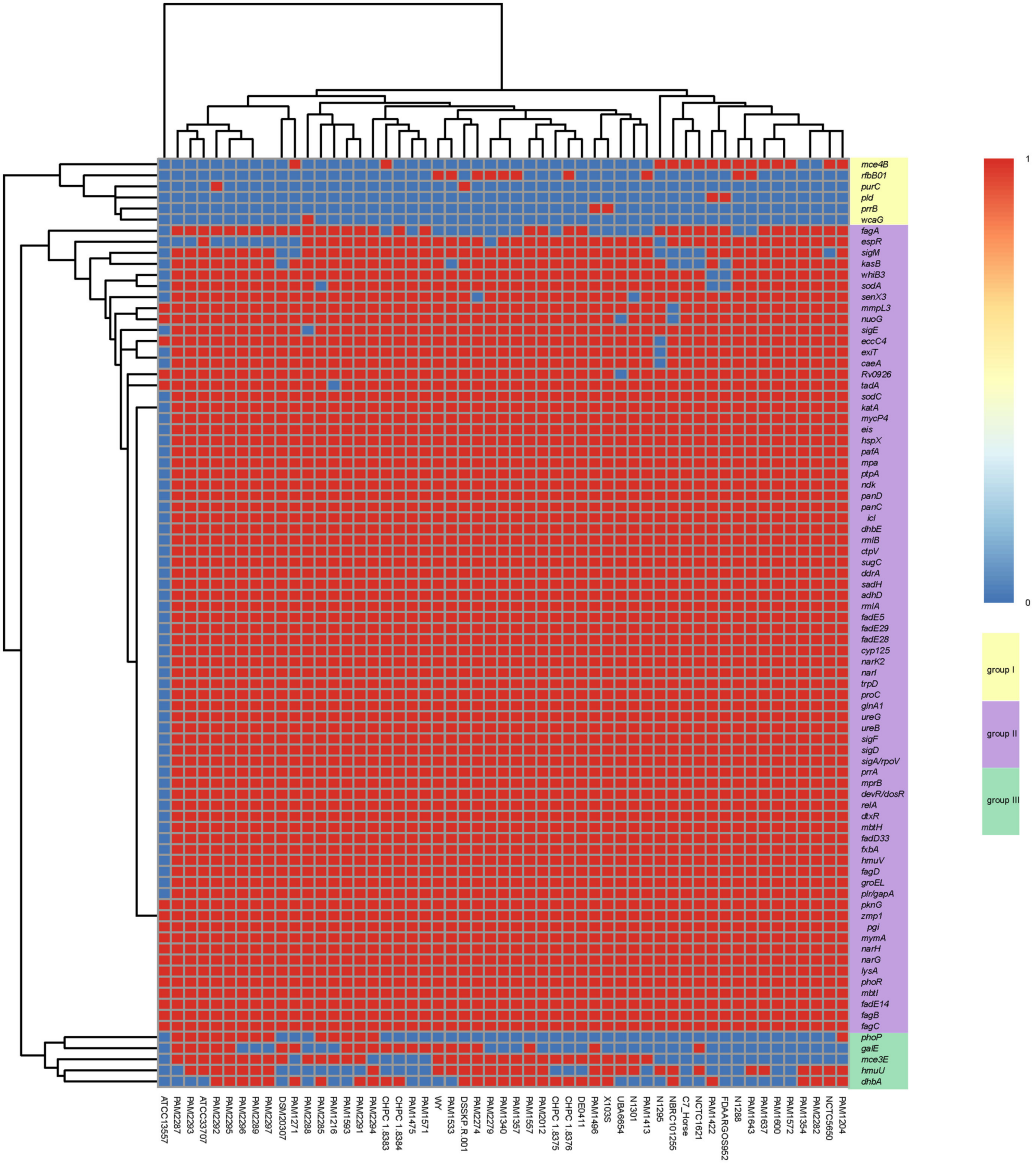
Heat map of virulence gene distribution in 53 R. equi strains. The predicted virulence genes were shown on the right. The strain names were shown at the bottom. The red color represented the presence of genes, while the blue color represented the absence.

A total of 84 virulence genes were predicted in the 53 *R. equi* strains. These virulence genes can be clustered into three groups (**Figure 5**). All strains except for ATCC13557 carried the majority of virulence genes in group II, followed by group III and Group I. These strains had almost the same virulence gene distribution except for *PhoP, gale, mce3e, hmuu, dhba, mce4b, rfbb01, purc, PLD, PrrB, WCAG* and *FAGA*. ATCC13557 had the least number of virulence genes. The virulence gene distribution was not clearly related to the host source and isolation region.

The predicted virulence genes of the 4 clinical *R. equi* strains were similar to those of other *R. equi* strains. 78 virulence genes were found (**Table S4**). In addition to the above common virulence genes, strain CHPC 1.8376 contained *FAGA*, *rfbb*01 and *dhba* genes related to ABC transporter, polysaccharide capsule and copper exporter, respectively, while strain CHPC 1.8383 contained *dhba, gale* and *mce4b* genes related to copper metabolism, polysaccharide capsule and mammalian cell entry (MCE) operons, respectively. Strain CHPC 1.8384 contained *fagA, Dhba* and *gale* genes related to ABC transporter, copper metabolism and polysaccharide capsule. CHPC 1.8375 strain contained *dhba* gene related to copper exporter.

### Antimicrobial Resistance Genes Analysis and *In Vitro* Susceptibility Testing

Six drug resistance-related genes were predicted in all 53 *R. equi* strains: *RbpA, tetA (33), erm(46), sul1, qacEdelta1* and *aadA9* (**Table 1**). *RbpA* gene is related to rifamycin resistance. Its product belongs to bacterial RNA polymerase binding protein family and is the main target of rifamycin. *RbpA* gene was found in all 53 *R. equi* genomes, and the matching degree was completely consistent with the coverage. *TetA (33)* gene and *qacEdelta1* gene belong to the main promoter superfamily (MFS), which is one of the largest solubilizer transport units so far. *TetA (33)* gene is associated with tetracycline resistance, while *qacEdelta 1* gene is related to acridine dye, disinfecting agents and intercalating dyes resistance. *erm (46)* gene is related to erm 23S rRNA methyltransferase and resistance to macrolide, lincosamide and streptogramin. *Sul1* gene is related to resistance to sulfonamide, while *aadA9* gene is associated with aminoglycoside resistance. Especially, PAM2288 strain with *tetA (33)* gene, PAM2289 strain with *erm (46)* gene, and PAM2293, PAM2294, PAM2295, PAM2296, PAM2291 and PAM2292 strains with *erm (46), sul1, qacedelta1, aadA9 and tetA(33)* genes, are multidrug-resistant strains. Strains of other *Rhodococcus* spp. also carried *RbpA* gene, which is the only common drug resistance gene among strains from different *Rhodococcus* spp.

**Table 1:** Distribution of antimicrobial resistance gene of *R. equi*.

Gene	Antibiotics class	Identity (%)	Coverage (%)	No. strain	Name of strain
*RbpA*	rifamycin antibiotic	89.19	98.25	53	All
*tetA(33)*	tetracycline antibiotic	100	100	8	PAM2288,PAM2291,PAM2292,PAM2293,PAM2294,PAM2295,PAM2296,PAM2297
*erm(46)*	macrolide antibiotic, lincosamide antibiotic, streptogramin antibiotic	100	100	8	PAM2289,PAM2291,PAM2292,PAM2293,PAM2294,PAM2295,PAM2296,PAM2297
*sul1*	sulfonamide antibiotic	100	100	7	PAM2291,PAM2292,PAM2293,PAM2294,PAM2295,PAM2296,PAM2297
*qacEdelta1*	acridine dye, disinfecting agents and intercalating dyes	100	100	7	PAM2291,PAM2292,PAM2293,PAM2294,PAM2295,PAM2296,PAM2297
*aadA9*	Aminoglycoside antibiotic	100	100	7	PAM2291,PAM2292,PAM2293,PAM2294,PAM2295,PAM2296,PAM2297

The results of antibiotic drug sensitivity test showed that the four strains in this study were susceptible to amikacin, chloramphenicol, rifampicin, levofloxacin, ciprofloxacin, colistin, fosfomycin, meropenem, minocycline, moxifloxacin, tetracycline, and tigecycline, but resistant to aztreonam, cefazolin, nitrofurantoin, amoxicillin-clavulanate, ampicillin-sulbactam, cefepime, cefoperazone-sulbactam, cefoxitin, ceftazidime, ceftriaxone, cefuroxime, trimethoprim-sulfamethoxazole, gentamicin and imipenem.

### Plasmid Related Gene Analysis

**Figure 6:**
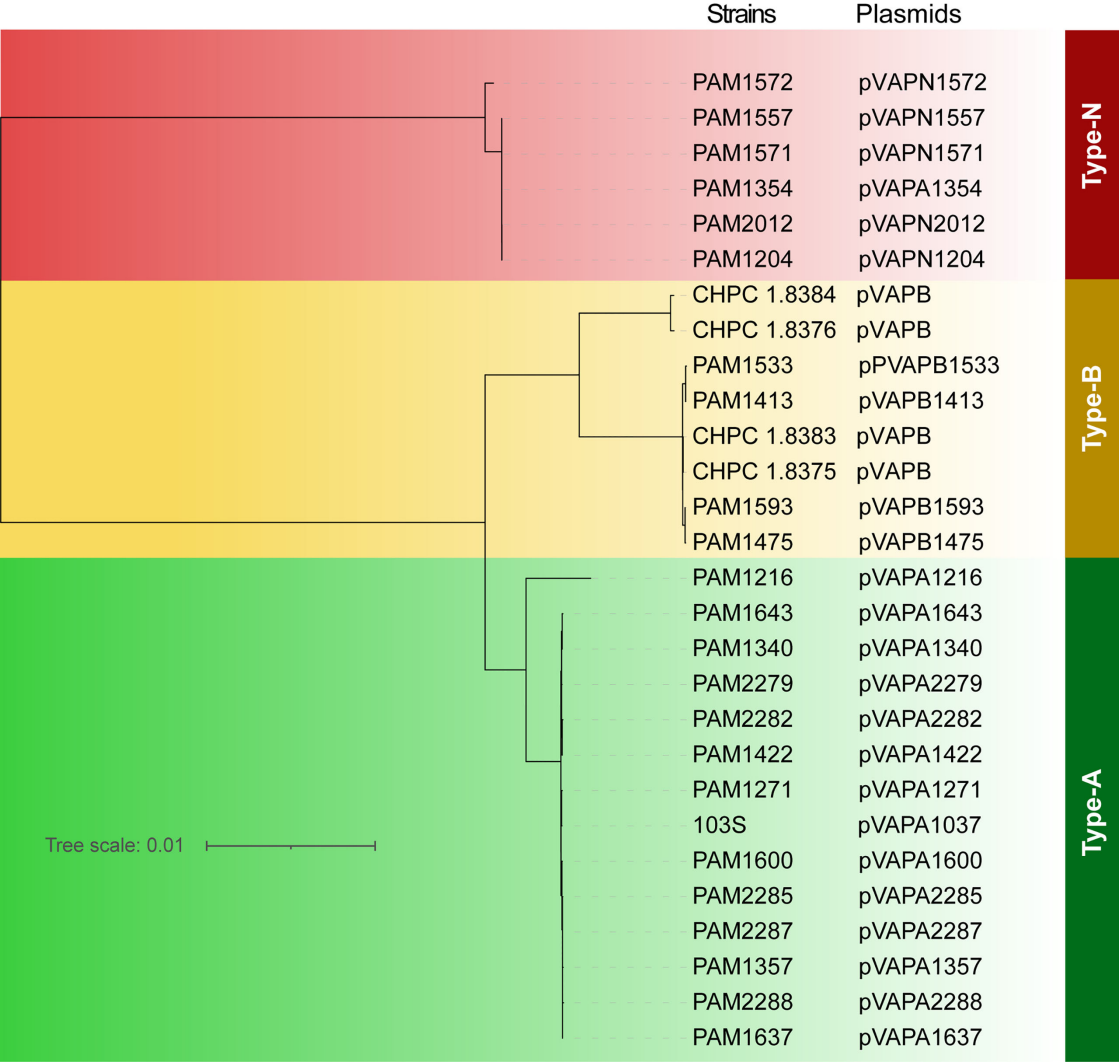
Phylogenetic tree based on full-length sequences of R. equi plasmids. The names of strains and plasmids were shown on the right. Different types of plasmids were labeled in different colors. Green for type-A plasmid, yellow for type-B plasmid, and red for type-N plasmid.

28 plasmids were found in the 53 *R. equi* strains. The evolutionary analysis based on pan-genome showed that the 28 plasmids can be divided into three types, type-A (n = 14), Type-B (n = 8) and type-N (n = 6) (**Figure 6**). The four *R. equi* strains in this study contained type-B plasmids. Notably, the plasmid pVAPN1354 (NCBI accession number: NZ_KX443399.1) from strain PAM1354 was previously considered as type-A, and this study corrected it to type-N.

**Figure 7:**
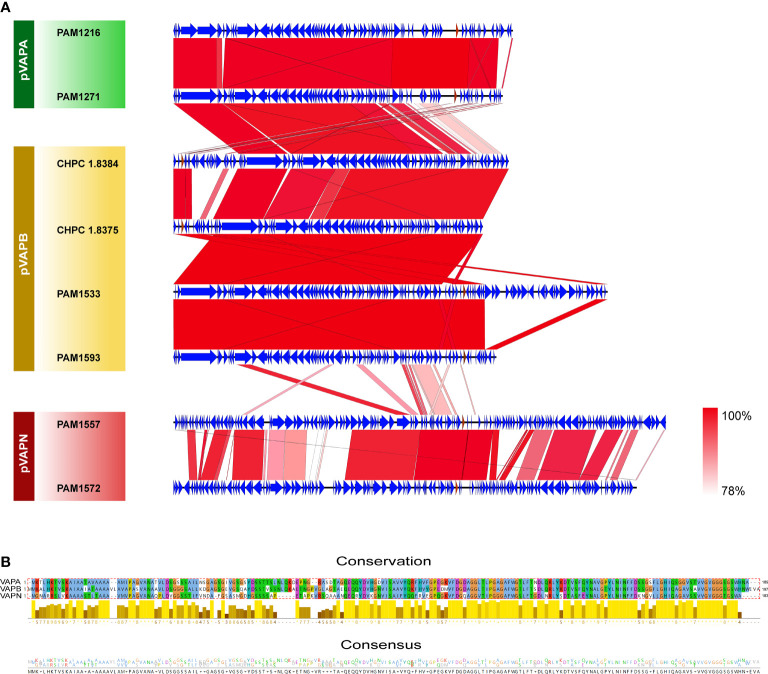
Genome comparisons of the host-associated virulence plasmids of *R.equi*. **(A)** Collinearity analysis of three VAP plasmids. The CDSs were represented by arrows, and the red arrows represented vapA/B/N gene. Regions with similar sequences were connected and labeled from pink to red. **(B)** Sequence comparison of the three types of VAP proteins. The alignment, conservation and consistency of amino acid sequences of the three types of VAP proteins were shown from the top to the bottom. Conserved regions were represented by histogram, in which bright colors and long bars represented highly conserved, while dark colors and short bars represented less conserved. At the bottom, the number of amino acids at each position represented the degree of consistency.

Due to the high homology among the genes of each plasmid type, we selected representative plasmid sequences for collinearity analysis. Genetic structures from the same plasmid type were highly similar. There was a high collinearity between plasmid type-A and type-B, while the collinearity between plasmid type-N and type-A/B was low. The variation between plasmids was mostly caused by scattered SNP and gene insertion rearrangement (**Figure 7A**). The VAP sequences from the three different plasmid types were type specific. The VAP genes were 100% consistent in the same type. The sequence similarities were 75.52% between type-A and type-B, 56.21% between type-A and type-N and 60.48% between type-B and type-N, respectively (**Figure 7B**).

## Discussion

Since *R. equi* was discovered from foals in 1923, it has become a well-known major equine pathogen in veterinary medicine. In humans, it mainly infects individuals with impaired immune function. HIV combined with *R. equi* infection often leads to serious clinical symptoms and high mortality. Although a number of veterinary studies have explained the pathogenic mechanisms of *R. equi* infection in animals, *R. equi* may infect humans by a different way and the studies of *R. equi* infection in humans are limited. In this study, the genomic characteristics, virulence factors and antibiotic resistance of 53 *R. equi* strains were explored by pan-genome analysis and comparative genomics to provide theoretical basis for the prevention and treatment of *R. equi* infection.

In our study, the genome of plasmid type-B *R. equi* isolated from HIV patients was determined for the first time, the average genome size of the four clinical isolates sequenced was 5.16M, average GC content was 68.57%, it is consistent with the general characteristics of *R. equi* genome. Previous studies have pointed out that the phylogenetic position of the species based on equivocal 16S rDNA and numerical phenetic studies has been repeatedly questioned, meanwhile, the ANI value of species well above the consensus 95-96% threshold for prokaryotic species demarcation (mean ANI value was 99.13%) (Guo et al., 2015; Li et al., 2015; Anastasi et al., 2016; Sangal et al., 2016). To more clearly identify the strains in this study, we collected the genomes of all *Rhodococcus* in the public database for ANI analysis. The results showed that the average ANI values of our four isolates were more than 98%, which was clearly identified as *R. equi*.

Pan-genomic analysis is used to explore the predicted gene pool, individual specific genes and accurate functional information of bacterial diversity. Our analysis showed that genes related to transport and metabolic system accounted for a high proportion in the whole genomes of 53 *R. equi* strains, which may suggest the effective transport of substrates and products. The proportions of genes related to transcription, translation, ribosomal structure, biosynthesis and metabolism were also high. These genes are important for cell growth and effective response to nutritional sources, which provides survival advantages in the changing environment. Similar to previous studies, these functions are related to the interactions between the bacteria and the environment, indicating that the genes may involve in niche adaptation and obtain through mobile genetic elements (Anastasi et al., 2016; MacArthur et al., 2017).

At present, the pathogenic mechanism of *R. equi* infection in humans is not clear. S Takai et al. believe that the toxic plasmids carried by *R. equi* are important for its pathogenicity (Takai et al., 2020). The toxic plasmids encode a group of virulence-related proteins, in which VAP and its homologous proteins are regarded as important virulence factors. The exact mechanism of VAP and its homologous proteins is still unknown, but it is considered to be related to the survival and replication of R. equi in macrophages (Geerds et al., 2021). Also, these plasmids are host-specific and can be found in both animals and humans. Therefore, the transmission source of R. equi in human infection can be inferred by the type of toxic plasmid contained. Previous studies about HIV and R. equi co-infection in humans showed that most of the R. equi strains contained type-B plasmids, suggesting that the exposure to pigs may be a major risk factor because type-B plasmids mostly come from pigs (Anastasi et al., 2016). Similarly, in our study, the four *R. equi* strains isolated from HIV patients contained type-B plasmids. Notably, the cluster analysis based on pan-genome showed that these four *R. equi* strains were closest to an environmental *R. equi* strain DSSKP-R-001, suggesting that environmental *R. equi* strains have the possibility to directly infect humans, but this inference still needs further verification because of the smaller number of strains from this study. Previous studies have shown that there was no obvious association between core–genome phylotypes and host source, whereas the latter was clearly linked with the host-associated plasmid type, no correlation between genomic types and the geographical origin of the isolates was observed (Anastasi et al., 2016). Our research basically supports the above view, the phylogenetic analysis based on Pan-genome showed that these strains were lack of directly related to the isolation location and host source, although some clusters can include most strains from the same location, such as Chinese cluster, the European cluster, and the USA cluster at the bottom of the tree. Although the *R. equi* strains analyzed in this study have geographical diversity, the sequences of the plasmids they contained were highly conserved. It should be noted that pVAPA1354 plasmid (NZ_KX443399.1) was labeled as type-A in GenBank, but in fact it should be classified as type-N. Because the plasmid sequences of other strains could not be obtained, the analysis of plasmids in the study only included 28 strains that could obtain plasmid information.

In order to further explore the pathogenic characteristics of *R. equi*, we annotated the genome of 53 *R. equi* strains by VFDB online database. Due to the lack of virulence factor library related to *Rhodococcus*, we used *Corynebacterium* as a reference for virulence gene analysis. 84 potential toxicity-related genes were obtained, showing rich diversity. Although the *R. equi* 53 *R. equi* strains can be preliminarily divided into four clusters according to the presence or absence of specific virulence genes, there was no significant difference among each cluster except for ATCC1355 strain, which only predicted to contain a few virulence genes and showed great differences from other strains. Genes related to secretory protein(*pknG)*, protease (*zmp1*), Immune evasion (*pgi*), Cell surface components (*mymA*), anaerobic respiration (*narH*, *narG*), amino acid and purine metabolism (*lysA*), regulation (*phoR*) and iron uptake *(mbtI*, *fadE14*, *fagB*, *fagC*) were found in all the studied strains. *pknG* gene was considered to play a core role in Rhocs, a newly discovered heavy oxygen home static system to regulate biofilm growth and host persistence in *mycobacteria* (Suzuki et al., 2021). *NarG* gene encodes nitrate reductase G, which participate in respiration under anaerobic conditions. The complete attenuation of virulence of *R. equi* with mutant *narG* in mice indicated that the full expression of *narG* may be important for the virulence in equine, and that the anaerobic or micro aerobic conditions may be important for the growth of *R. equi* during infection (Pei et al., 2007). Most strains contain *SOD* gene, the products of which contribute to the survival of *R. equi* in macrophages. *fbpB* gene was also found. In *Mycobacterium tuberculosis*, *fbpB* encodes Ag85B, which is the immunodominant component of *Mycobacterium* antigen 85 complex (Ag85), which is a secretory protein with transferase activity and participates in the biogenesis of cell wall (Karbalaei et al., 2019). However, some studies have shown that in *Streptococcus equi*, *fbpB* was not necessary for virulence. The mutation of *fbpB* also did not affect the survival rate of mice infected with *R. equi* (Rahman et al., 2005). Among the three mycosyl transferase genes of *Mycobacterium tuberculosis*, only the mutation of *fbpA* was considered to weaken the growth of this species in macrophages. *Fade14* gene was also predicted in this study, *FadE14* is part of an iron and IdeR repressible operon which produces a polycistronic message encoding FadE14, FadD33 and an acyl carrier protein (acp), in *Mycobacterium smegmatis*, FadE14 were necessary for wild-type mycobactin production (Rodgers et al., 2021). The ability to metabolize fatty acids seems to be crucial to the toxicity of *Streptococcus equi*. A recent study showed that *fadd13*, a chain fatty acid CoA ligase related gene, promoted the growth of *R. equi* in macrophages. The virulence genes predicted in this study need follow-up experiments to validate the association with pathogenicity of *R. equi*. Also, we lack biological information to effectively detect the relations between virulence-related genes and the isolation sources of *R. equi*. The pathogenicity of *R. equi* may be affected by many factors, considering the complex interactions between bacteria and the environment.

In the past few years, various investigations have reported a wide range of drug-resistant *R. equi* strains from environmental and clinical samples, including resistance to macrolides, lincomides, streptomycin, tetracycline and rifampicin. The study of Sonsiray Álvarez-Narváez demonstrates that the increasing prevalence of multidrug-resistant (MDR) *R. equi* since its emergence in the late 1990s-early 2000s in equine farms in the United States, MDR *R. equi* shows resistance to several clinically relevant antibiotic drugs, including macrolides, lincosamides, streptomycin and tetracycline resistance by the *pRErm46* conjugative plasmid; and rifampicin conferred by a chromosomal *rpoB* mutation (Alvarez-Narvaez et al., 2021a). In our study, various genetic elements related to drug resistance were found in all 53 *R. equi* strains. These genes were shown to be resistant to different kinds of antibiotics, including rifamycin, macrolide, lincomamide, streptomycin and sulfa. In addition to the *Erm46* gene mentioned in the above study, we also predicted the *tetA (33)* gene related to tetracycline antibiotics; *sul1* gene related to sulfonamide antibiotic resistance and *aadA9* gene related to aminoglycoside antibiotic. Rifampin resistance in *R. equi* results from mutations in the beta subunit of the RNA polymerase (*rpoB*) gene, substitutions that confer rifampicin resistance were resulted in a decreased affinity for rifampicin. So far, the rifampicin resistance of *R. equi* has been related to *rpoB* gene (Alvarez-Narvaez et al., 2021; Alvarez-Narvaez et al., 2021a). However, we only predicted the *RbpA* gene associated with rifampicin resistance (matching region was 89%) in the four strains in this study, and not associated with phenotype. RbpA is an 14 kDa RNA polymerase (RNAP)-binding protein whose presence increases the tolerance levels of *Mycobacteria* to rifampicin by driven stimulation of the housekeeping gene expression, widely exists in actinomycetes, the RbpA-driven stimulation of the housekeeping gene expression may help *Mycobacteria* to tolerate high rifampicin levels and to adapt to the stress conditions during infection (Prusa et al., 2018; Torfs et al., 2019).

## Conclusion

Our findings characterized four clinical *R. equi* strains isolated from HIV patients in China. These strains are closely related to several *R. equi* strains isolated from environment, suggesting that the *R. equi* infection in the HIV patients may be environmental. Through comparative genomics, we identified new potential virulence genes and drug resistance genes in *R. equi*. These genes may play an important role in the evolution and virulence of *R. equi*. Our study provides important insights into the epidemic and pathogenic mechanism of *R. equi*, which also provided a theoretical basis for the prevention and control of infectious diseases caused by *R. equi*.

## Data Availability Statement

The datasets presented in this study can be found in online repositories. The names of the repository/repositories and accession number(s) can be found below: NCBI; PRJNA773582.

## Ethics Statement

This study used strains obtained from clinical sources and the Institutional human Ethics Committee of the Institute did not require the study to be reviewed or approved because the strains were from laboratory stock. Written informed consent for participation was not required for this study in accordance with the national legislation and the institutional requirements. The data were analyzed anonymously and reported.

## Author Contributions

YW, DW and QW designed the work. YS, XX, ZH and KY performed the experiments. MJ, SY, MZ, HM and YH collected samples and isolated strains. YS, ZH, KY, YX and QW analyzed the data. YS, ZH, YX and DW wrote the manuscript. All authors contributed to the article and approved the submitted version.

## Funding

This work was supported by National Science and Technology Major Project on Important Infectious Diseases Prevention and Control (No.2018ZX10734404), the National Science and Technology Infrastructure of China (National Pathogen Resource Center-NPRC-32 and 2021FY100900).

## Conflict of Interest

The authors declare that the research was conducted in the absence of any commercial or financial relationships that could be construed as a potential conflict of interest.

The reviewer QW declared a shared affiliation with several of the authors, SY, ZH, YX, KY, MJ, DW, and QW, to the handling editor at time of review.

## Publisher’s Note

All claims expressed in this article are solely those of the authors and do not necessarily represent those of their affiliated organizations, or those of the publisher, the editors and the reviewers. Any product that may be evaluated in this article, or claim that may be made by its manufacturer, is not guaranteed or endorsed by the publisher.
