# Corrigendum: Genomic Characteristics and Pan-Genome Analysis of *Rhodococcus equi*

**DOI:** 10.3389/fcimb.2022.884441

**Published:** 2022-04-12

**Authors:** Yang Song, Xinmin Xu, Zhenzhou Huang, Yue Xiao, Keyi Yu, Mengnan Jiang, Shangqi Yin, Mei Zheng, Huan Meng, Ying Han, Yajie Wang, Duochun Wang, Qiang Wei

**Affiliations:** ^1^ National Pathogen Resource Center (NPRC), Chinese Center for Disease Control and Prevention (China CDC), Beijing, China; ^2^ Department of Clinical Laboratory, Beijing Ditan Hospital, Capital Medical University, Beijing, China; ^3^ Center for Human Pathogenic Culture Collection (CHPC), National Institute for Communicable Disease Control and Prevention (ICDC), China CDC, Beijing, China

**Keywords:** genomic characteristics, plasmid-mediated pathogenicity, rifamycin resistance, *Rhodococcus equi*, pan-genome

In the original article, there was an error in the title. The study does not directly reveal plasmid-mediated pathogenicity or ubiquitous rifamycin resistance.

The title has been corrected as follows, “Genomic Characteristics and Pan-genome Analysis of *Rhodococcus equi”*.

The authors apologize for this error and state that this does not change the scientific conclusions of the article in any way. The original article has been updated.

## Publisher’s Note

All claims expressed in this article are solely those of the authors and do not necessarily represent those of their affiliated organizations, or those of the publisher, the editors and the reviewers. Any product that may be evaluated in this article, or claim that may be made by its manufacturer, is not guaranteed or endorsed by the publisher.

