# Supplementary material for: Genomic Characteristics and Pan-Genome Analysis of Rhodococcus equi
Source: Front Cell Infect Microbiol. 2022 Feb 16;12:807610. doi: 10.3389/fcimb.2022.807610 (PMC8891757; doi:10.3389/fcimb.2022.807610)
Supplement: Supplementary file 1 [file DataSheet_1.pdf]

Table S1 Detailed information of 53 *R.equi* genomes.

No.	Strain	Size (Mb)	G+C(mol%)	CDS	tRNA	rRNA	tmRNA	Level	Accession No.	Plasmid type	Plasmid accession No.
1	CHPC 1.8375*	5.13	68.52	4778	61	7	1	Contig	JAJENO000000000	VAPB	
2	CHPC 1.8376*	5.26	68.61	4937	66	8	1	Contig	JAJENN000000000	VAPB	
3	CHPC 1.8383*	5.08	68.57	4710	60	8	1	Contig	JAJENL000000000	VAPB	
4	CHPC 1.8384*	5.16	68.56	4796	61	3	1	Contig	JAJENL000000000	VAPB	
5	103S	5.04	68.8	4582	63	12	1	Complete	GCA_000196695.1	VAPA	NC_011151.1
6	ATCC 13557	5.29	68.5	4563	66	5	1	Complete	GCA_016921075.1	-	-
7	ATCC 33707	5.26	68.75	4898	64	15	1	Contig	GCA_000164155.2	-	-
8	C 7	5.2	68.8	4742	62	3	NA	Contig	GCA_000473915.1	-	-
9	DE0411	5.27	68.8	4819	63	3	1	Scaffold	GCA_007672705.1	-	-
10	DSM 20307	5.2	68.8	4772	64	3	1	Contig	GCA_002094305.1	-	-
11	DSSKP-R-001	5.44	68.66	5055	75	15	1	Complete	GCA_003013675.1	-	-
12	FDAARGOS_952	5.22	68.8	4768	63	12	1	Complete	GCA_016025875.1	-	-
13	N1288	5.17	68.8	4737	59	3	1	Contig	GCA_001646885.1	-	-
14	N1295	5.31	68.7	4869	64	3	1	Contig	GCA_001646905.1	-	-
15	N1301	5.65	68.5	5179	60	3	1	Contig	GCA_001646925.1	-	-
16	NBRC 101255	5.2	68.8	4771	63	3	1	Contig	GCA_001552575.1	-	-
17	NCTC1621	5.24	68.8	4783	64	12	2	Contig	GCA_900455845.1	-	-
18	NCTC5650	5.31	68.7	4820	63	12	1	Contig	GCA_900455885.1	-	-
19	PAM1204	5.23	68.8	4773	61	3	1	Contig	GCA_002078545.1	VAPN	NZ_KX443398.1
20	PAM1216	5.2	68.8	4772	67	3	1	Contig	GCA_002095175.1	VAPA	NZ_KX443388.1
21	PAM1271	5.2	68.8	4780	66	3	1	Contig	GCA_002095045.1	VAPA	NZ_KX443396.1

22	PAM1340	5.06	68.8	4633	61	3	1	Contig	GCA_002095085.1	VAPA	NZ_KX443392.1
23	PAM1354	5.37	68.7	4919	67	3	1	Contig	GCA_002095035.1	VAPN	NZ_KX443399.1
24	PAM1357	5.01	68.8	4578	63	3	1	Contig	GCA_002095125.1	VAPA	NZ_KX443389.1
25	PAM1413	5.04	68.8	4606	64	3	1	Contig	GCA_002095115.1	VAPB	NZ_KX443406.1
26	PAM1422	5.2	68.8	4780	64	3	1	Contig	GCA_002095195.1	VAPA	NZ_KX443390.1
27	PAM1475	5.21	68.8	4768	63	4	1	Contig	GCA_002095185.1	VAPB	NZ_KX443397.1
28	PAM1496	5.09	68.8	4651	63	3	1	Contig	GCA_002095155.1	-	-
29	PAM1533	5.13	68.8	4722	62	3	1	Contig	GCA_002095235.1	VAPB	NZ_KX443407.1
30	PAM1557	5.35	68.7	4908	69	3	1	Contig	GCA_002095255.1	VAPN	NZ_KX443400.1
31	PAM1571	5.42	68.6	4951	65	3	1	Contig	GCA_002094265.1	VAPN	NZ_KF439868.1
32	PAM1572	5.12	68.8	4669	61	3	1	Contig	GCA_002078535.1	VAPN	NZ_KX443401.1
33	PAM1593	5.24	68.7	4807	62	3	1	Contig	GCA_002078625.1	VAPB	NC_011150.1
34	PAM1600	5.14	68.8	4703	59	3	1	Contig	GCA_002078515.1	VAPA	NZ_KX443391.1
35	PAM1637	5.08	68.9	4634	66	3	1	Contig	GCA_002095295.1	VAPA	NZ_KX443393.1
36	PAM1643	5.09	68.8	4691	62	3	1	Contig	GCA_002094235.1	VAPA	NZ_KX443394.1
37	PAM2012	5.33	68.7	4877	66	3	1	Contig	GCA_002094445.1	VAPN	NZ_KP851975.1
38	PAM2274	5.22	68.7	4812	60	3	1	Contig	GCA_002094225.1	-	-
39	PAM2279	5.03	68.8	4619	61	3	1	Contig	GCA_002094375.1	VAPA	NZ_KX443405.1
40	PAM2282	5.27	68.7	4831	63	3	1	Contig	GCA_002094295.1	VAPA	NZ_KX443395.1
41	PAM2285	5.19	68.8	4746	64	3	1	Contig	GCA_002094395.1	VAPA	NZ_KX443402.1
42	PAM2287	5.14	68.9	4684	63	3	1	Contig	GCA_002094405.1	VAPA	NZ_KX443404.1
43	PAM2288	5.48	68.7	5081	65	3	1	Contig	GCA_002094325.1	VAPA	NZ_KX443403.1
44	PAM2289	5.35	68.7	4916	62	3	1	Contig	GCA_017116975.1	-	-
45	PAM2291	5.47	68.7	5040	62	3	1	Contig	GCA_017117025.1	-	-
46	PAM2292	5.45	68.6	5071	66	3	1	Contig	GCA_017117065.1	-	-
47	PAM2293	5.4	68.7	4999	65	3	1	Contig	GCA_017117085.1	-	-

48	PAM2294	5.41	68.6	4963	64	2	1	Scaffold	GCA_017117125.1	-	-
49	PAM2295	5.41	68.7	4998	64	3	1	Contig	GCA_017117005.1	-	-
50	PAM2296	5.4	68.7	5002	63	3	1	Contig	GCA_017117015.1	-	-
51	PAM2297	5.35	68.7	4922	62	3	1	Contig	GCA_017116965.1	-	-
52	UBA6654	5.26	68.2	5058	63	NA	1	Scaffold	GCA_002455495.1	-	-
53	WY	5.21	68.73	4751	66	15	1	Complete	GCA_007197835.1	-	-

*Strains sequenced in this study

Table S2 Detailed information of 33 *R.*genome.

No.	Organism Name	Strain	Size(Mb)	G+C(mol%)	Level	Accession No.
1	*R.aetherivorans*	BCP1	6.23	70.34	complete	GCA_000470885.1
2	*R.agglutinans*	CCTCCAB2014297	5.43	69.2	contig	GCA_004011865.1
3	*R.biphenylivorans*	TG9	5.03	68	complete	GCA_003288095.1
4	*R.coprophilus*	NCTC10994	4.58	66.8	complete	GCA_900478115.1
5	*R.corynebacterioides*	NBRC14404	3.97737	70.3	contig	GCA_001894765.1
6	*R.enclensis*	NIO-1009	7.47874	62.3	scaffold	GCA_900094765.1
7	*R.erythropolis*	NCTC8036	6.55124	62.4	contig	GCA_900455855.1
8	*R.fascians*	D188	5.50211	64.584	complete	GCA_001620305.1
9	*R.globerulus*	WS3306	6.77108	61.7	contig	GCA_003097035.1
10	*R.gordoniae*	NCTC13296	4.87192	67.9	contig	GCA_900455725.1
11	*R.hoagii*	DSSKP-R-001	5.43883	68.6647	complete	GCA_003013675.1
12	*R.imtechensis*	RKJ300	8.23134	67.2	contig	GCA_000260815.1
13	*R.jostii*	DSM44719	9.91298	66.9	contig	GCA_900105375.1
14	*R.koreensis*	DSM44498	10.3116	67.4	contig	GCA_900105905.1
15	*R.kroppenstedtii*	DSM44908	4.08283	70.1	contig	GCA_900111805.1
16	*R.kyotonensis*	JCM23211	6.31101	64.2	scaffold	GCA_900188125.1

17	*R.maanshanensis*	NBRC100610	5.67371	69.2	contig	GCA_001894865.1
18	*R.marinonascens*	NBRC14363	4.92	64.4	contig	GCA_001894885.1
19	*R.opacus*	DSM44186	8.84147	67.1027	complete	GCA_019856255.1
20	*R.oryzae*	NEAU-CX67	5.37	69.2	contig	GCA_005049235.1
21	*R.percolatus*	WS3313	7.88481	67.4	contig	GCA_017875695.1
22	*R.pyridinivorans*	DSM44555	5.26248	67.8	contig	GCA_900105195.1
23	*R.qingshengii*	CS98	6.72611	62.3484	complete	GCA_015099595.1
24	*R.rhodnii*	ATCC35071	4.4918	69.6	scaffold	GCA_008011915.1
25	*R.rhodochrous*	NCTC10210	5.27476	68.2	complete	GCA_900187265.1
26	*R.ruber*	SD3	5.36661	70.6	complete	GCA_003086595.1
27	*R.subtropicus*	C9-28	4.4	69.1	scaffold	GCA_005434945.1
28	*R.triatomae*	DSM44893	4.77616	68.7	complete	GCA_014217765.1
29	*R.trifolii*	CCM7905	5.29	65.5	contig	GCA_014635345.1
30	*R.tukisamuensis*	JCM11308	5.48901	69.8	contig	GCA_900101735.1
31	*R.wratislaviensis*	WS3308	7.83569	67.4	contig	GCA_003385055.1
32	*R.yunnanensis*	NBRC103083	6.37	63.9	contig	GCA_001895005.1
33	*R.zopfii*	NBRC100606	6.29721	68.2	contig	GCA_001895025.1

Table S3 Antimicrobial resistance gene of *Rhodococcus*

Name	Gene	NO.of Strain	Drug Class	Identity of Matching Region %	Coverage of Reference Sequence %
R.agglutinans	*RbpA*	1	rifamycin antibiotic	90.99	98.25
R.biphenylivorans	*RbpA*	1	rifamycin antibiotic	89.19	98.25
R.coprophilus	*RbpA*	1	rifamycin antibiotic	88.29	98.25

R.corynebacterioides	*RbpA*	1	rifamycin antibiotic	86.73	100
R.enclensis	*RbpA*	1	rifamycin antibiotic	86.49	98.25
	Trimethoprim-resistant dihydrofolate reductase DfrA43	1	diaminopyrimidine antibiotic	39.71	83.59
	iri	1	rifamycin antibiotic	95.79	99.79
	Mycobacterium tuberculosis folC with mutation conferring resistance to para-aminosalicylic acid	1	para-aminosalicylic acid	67.77	98.36
R.erythropolis	*iri*	1	rifamycin antibiotic	96.19	99.16
	RbpA	1	rifamycin antibiotic	85.59	98.25
	Mycobacterium tuberculosis folC with mutation conferring resistance to para-aminosalicylic acid	1	para-aminosalicylic acid	67.77	98.36
R.fascians D188		1	antibiotic target protection	98.25	
R.globerulus	*RbpA*	1	rifamycin antibiotic	86.49	98.25
	Trimethoprim-resistant dihydrofolate reductase DfrA43	1	diaminopyrimidine antibiotic	40.14	83.59
	Mycobacterium tuberculosis folC with mutation conferring resistance to para-aminosalicylic acid	1	para-aminosalicylic acid	67.63	99.38
R.gordoniae	*RbpA*	1	rifamycin antibiotic	89.19	98.25
R.imtechensis RKJ300	*RbpA*	1	rifamycin antibiotic	88.29	98.25
	Mycobacterium tuberculosis folC with mutation conferring resistance to para-aminosalicylic acid	1	para-aminosalicylic acid	68.72	97.54
R.jostii	*RbpA*	1	rifamycin antibiotic	87.39	98.25
R.koreensis	Trimethoprim-resistant dihydrofolate reductase DfrA43	1	diaminopyrimidine antibiotic	33.72	83.59

	RbpA	1	rifamycin antibiotic	88.29	98.25
R.kroppenstedtii	*RbpA*	1	rifamycin antibiotic	87.61	100
R.kyotonensis	*RbpA*	1	rifamycin antibiotic	90.09	98.25
R.maanshanensis	*RbpA*	1	rifamycin antibiotic	88.29	98.25
R.marinonascens	*RbpA*	1	rifamycin antibiotic	87.39	98.25
	Mycobacterium tuberculosis folC with mutation conferring resistance to para-aminosalicylic acid	1	para-aminosalicylic acid	66.6	97.54
R.opacus	Trimethoprim-resistant dihydrofolate reductase DfrA43	1	diaminopyrimidine antibiotic	33.72	86.15
	RbpA	1	rifamycin antibiotic	88.29	98.25
	Mycobacterium tuberculosis folC with mutation conferring resistance to para-aminosalicylic acid	1	para-aminosalicylic acid	68.51	97.54
R.oryzae	Trimethoprim-resistant dihydrofolate reductase DfrA43	1	diaminopyrimidine antibiotic	37.72	82.56
	vanRO	1	glycopeptide antibiotic	99.57	100
	vanXO	1	glycopeptide antibiotic	95.05	100
	vanO	1	glycopeptide antibiotic	97.11	100
	vanHO	1	glycopeptide antibiotic	96.86	100
	RbpA	1	rifamycin antibiotic	88.29	98.25
R.percolatus	*RbpA*	1	rifamycin antibiotic	88.29	98.25
	Trimethoprim-resistant dihydrofolate reductase DfrA43	1	diaminopyrimidine antibiotic	33.72	86.15

	Mycobacterium tuberculosis folC with mutation conferring resistance to para-aminosalicylic acid	1	para-aminosalicylic acid	68.72	97.54
R.pyridinivorans	*RbpA*	1	rifamycin antibiotic	89.19	98.25
R.qingshengii	*RbpA*	1	rifamycin antibiotic	86.49	98.25
	Mycobacterium tuberculosis folC with mutation conferring resistance to para-aminosalicylic acid	1	para-aminosalicylic acid	67.77	98.36
R.rhodnii	*RbpA*	1	rifamycin antibiotic	85.59	98.25
R.rhodochrous	*RbpA*	1	rifamycin antibiotic	88.29	98.25
R.ruber	Trimethoprim-resistant dihydrofolate reductase DfrA43	1	diaminopyrimidine antibiotic	33.15	84.62
	RbpA	1	rifamycin antibiotic	84.68	98.25
R.sp. BCP1	Trimethoprim-resistant dihydrofolate reductase DfrA43	1	diaminopyrimidine antibiotic	32.02	84.62
	RbpA	1	rifamycin antibiotic	84.68	98.25
R.subtropicus	*RbpA*	1	rifamycin antibiotic	88.29	98.25
R.triatomae	*RbpA*	1	rifamycin antibiotic	87.39	98.25
R.trifolii	*RbpA*	1	rifamycin antibiotic	87.72	100
R.tukisamuensis	*RbpA*	1			
R.wratislaviensis	*RbpA*	1	rifamycin antibiotic	88.29	98.25
	Trimethoprim-resistant dihydrofolate reductase DfrA43	1	diaminopyrimidine antibiotic	32.74	86.15
	Mycobacterium tuberculosis folC with mutation conferring resistance to para-aminosalicylic acid	1	para-aminosalicylic acid	68.72	97.54
R.yunnanensis	*RbpA*	1	rifamycin antibiotic	88.29	98.25
R.zopfii	*RbpA*	1	rifamycin antibiotic	86.49	98.25

Table S4 The virulence factors of *R.equi*.

VFclass	Virulence factors	Related genes
Adherence	GroEL(*Clostridium*)	*groEL*
	Streptococcal plasmin receptor/GAPDH(*Streptococcus*)	*plr/gapA*
	The tad locus(*Haemophilus*)	*tadA*
Iron uptake	ABC transporter	*fagA*
		fagB
		fagC
		fagD
		hmuU
		hmuV
	Exochelin (smegmatis)(*Mycobacterium*)	*exiT*
		fxbA
	Heme uptake(*Mycobacterium*)	*mmpL3*
	Mycobactin(*Mycobacterium*)	*fadD33*
		fadE14
		mbtH
		mbtI
Regulation	Diphtheria toxin repressor DtxR	*dtxR*
	(p)ppGpp synthesis and hydrolysis(*Mycobacterium*)	*relA*
	DevR/S(*Mycobacterium*)	*devR/dosR*
	MprA/B(*Mycobacterium*)	*mprB*
	PhoP/R(*Mycobacterium*)	*phoP*
		phoR
	PrrA/B(*Mycobacterium*)	*prrA/B*

	SenX3(*Mycobacterium*)	*senX3*
	Sigma A(*Mycobacterium*)	*sigA/rpoV*
	Sigma D(*Mycobacterium*)	*sigD*
	Sigma E(*Mycobacterium*)	*sigE*
	Sigma F(*Mycobacterium*)	*sigF*
	Sigma M(*Mycobacterium*)	*sigM*
	WhiB3(*Mycobacterium*)	*whiB3*
Toxin	Phospholipase D	*pld*
Acid resistance	Urease(Helicobacter)	*ureB*
		ureG
Amino acid and purine metabolism	Glutamine synthesis(*Mycobacterium*)	*glnA1*
	Lysine synthesis(*Mycobacterium*)	*lysA*
	Proline synthesis(*Mycobacterium*)	*proC*
	Purine synthesis(*Mycobacterium*)	*purC*
	Tryptophan synthesis(*Mycobacterium*)	*trpD*
Anaerobic respiration	Nitrate reductase(*Mycobacterium*)	*narG*
		narH
		narI
	Nitrate/nitrite transporter(*Mycobacterium*)	*narK2*
Anti-apoptosis factor	NuoG(*Mycobacterium*)	*nuoG*
Catabolism of cholesterol	Cyp125(*Mycobacterium*)	*cyp125*
	FadE28(*Mycobacterium*)	*fadE28*
	FadE29(*Mycobacterium*)	*fadE29*
Cell surface components	Carboxylesterase(*Mycobacterium*)	*caeA*
	GPL locus(*Mycobacterium*)	*Rv0926*
		fadE5

		rmlA
		adhD
	MymA operon(*Mycobacterium*)	*mymA*
		sadH
	PDIM (phthiocerol dimycocerosate) and PGL (phenolic glycolipid) biosynthesis and transport(*Mycobacterium*)	*ddrA*
	Trehalose-recycling ABC transporter(*Mycobacterium*)	*sugC*
Copper uptake	Copper exporter(*Mycobacterium*)	*ctpV*
Immune evasion	Capsule(*Streptococcus*)	*rmlB/rfbB-1*
	Exopolysaccharide(*Haemophilus*)	*pgi*
	Polysaccharide capsule(*Bacillus*)	*galE*
Iron acquisition	Bacillibactin(*Bacillus*)	*dhbA/E*
Lipid and fatty acid metabolism	FAS-II(*Mycobacterium*)	*kasB*
	Isocitrate lyase(*Mycobacterium*)	*icl*
	Pantothenate synthesis(*Mycobacterium*)	*panC*
		panD
Mammalian cell entry (mce) operons	Mce	*mce3E/mce4B*
Others	O-antigen(*Yersinia*)	*wcaG*
Phagosome arresting	Nucleoside diphosphate kinase(*Mycobacterium*)	*ndk*
	Tyrosine phosphatase(*Mycobacterium*)	*ptpA*
Protease	Proteasome-associated proteins(*Mycobacterium*)	*mpa*
		pafA
	Zn++ metallophrotease(*Mycobacterium*)	*zmp1*
Secreted proteins	Alpha-crystallin(*Mycobacterium*)	*hspX*
	Enhanced intracellular survival protein(*Mycobacterium*)	*eis*
	Protein kinase G(*Mycobacterium*)	*pknG*

Secretion system	ESX-1 (T7SS)(*Mycobacterium*)<br>ESX-4 (T7SS)(*Mycobacterium*)	*espR*<br>*eccC4*<br>*mycP4*
Stress adaptation	Catalase(*Neisseria*)<br>Cu<br>Iron-cofactored SOD(*Mycobacterium*)	*katA*<br>*sodC*<br>*sodA*
